# Perceptions, attitudes, and behaviors among adolescents living with obesity, caregivers, and healthcare professionals in Italy: the ACTION Teens study

**DOI:** 10.1007/s40519-024-01663-7

**Published:** 2024-05-08

**Authors:** Claudio Maffeis, Luca Busetto, Malgorzata Wasniewska, Daniele Di Pauli, Carla Maccora, Andrea Lenzi

**Affiliations:** 1 Department of Surgery, Dentistry, Gynecology and Pediatrics, Section of Pediatric Diabetes and Metabolism, University of Verona, Verona, Italy; 2 Department of Medicine, University of Padova, Padova, Italy; 3 Department of Human Pathology in Adulthood and Childhood, University of Messina, Messina, Italy; 4 Rovereto, Italy; 5 Novo Nordisk, Rome, Italy; 6 Department of Experimental Medicine, Section of Pathophysiology, Endocrinology and Food Sciences, University of Rome La Sapienza, Rome, Italy

**Keywords:** Adolescents, Obesity, Physician attitudes, Weight-loss motivators, Communication barriers

## Abstract

**Purpose:**

ACTION Teens (NCT05013359) was conducted in 10 countries to identify perceptions, attitudes, behaviors, and barriers to effective obesity care among adolescents living with obesity (ALwO), caregivers of ALwO, and healthcare professionals (HCPs). Here, we report data from participants in Italy.

**Methods:**

The ACTION Teens cross-sectional online survey was completed by 649 ALwO (aged 12– < 18 years), 455 caregivers, and 252 HCPs in Italy in 2021.

**Results:**

Most ALwO thought their weight was above normal (69%), worried about weight affecting their future health (87%), and reported making a weight-loss attempt in the past year (60%); fewer caregivers responded similarly regarding their child (46%, 72%, and 33%, respectively). In addition, 49% of caregivers believed their child would lose excess weight with age. ALwO (38%) and caregivers (30%) most often selected wanting to be more fit/in better shape as a weight-loss motivator for ALwO; HCPs most often selected improved social life/popularity (73%). ALwO (25%) and caregivers (22%) most frequently selected lack of hunger control and not liking exercise, respectively, as weight-loss barriers, while HCPs most often agreed that unhealthy eating habits were a barrier (93%). ALwO most often obtained weight-management information from family/friends (25%) and search engines (24%); caregivers most often obtained information from doctors (29%).

**Conclusion:**

In Italy, the impact of obesity on ALwO was underestimated by caregivers, and ALwO and HCPs had different perceptions of key weight-loss motivators and barriers. Additionally, the internet was a key information source for ALwO, which suggests new education/communication strategies are needed.

**Level of evidence:**

IV; Evidence obtained from multiple time series with/without intervention, e.g. case studies.

*Trial Registration:* ClinicalTrials.gov, NCT05013359.

## Introduction

Childhood obesity has a high prevalence in Italy (21% in boys and 14% in girls [aged 7–9 years]), as in most Southern European countries [1]. Excess adiposity in youth has several worrisome consequences, including the high persistence of obesity into adulthood (up to 80% of cases) and the association with increased morbidity and mortality in later life [2]. Youths with obesity can experience impaired health-related quality of life, discrimination, and stigma, which can lead to unhealthy eating behaviors, binge eating, and a reluctance to participate in physical activity, with the risk of worsening their obesity. Additionally, obesity-related stigma can increase vulnerability to depression, anxiety, low self-esteem, body image disturbance, and suicidal ideation [3].

As such, there is an urgent need for early diagnosis and treatment of obesity in children and adolescents, especially in light of evidence demonstrating that long-term outcomes (including cardiometabolic risk) are better for adolescents living with obesity (ALwO) when they receive early intervention [4, 5]. However, parents and caregivers frequently underestimate the overweight status of their children [6]. This may lead to a delayed obesity diagnosis, which may in turn delay the initiation of treatment of obesity and any complications that are already present.

Healthcare professionals (HCPs) play an important role in the assessment and treatment of obesity in children/adolescents [7], therefore their involvement is crucial in obesity care. Three factors should be considered for improving HCP activity in this field: (i) their level of familiarity with the guidelines for obesity diagnosis and management; (ii) the potential barriers to effective communication with ALwO and their caregivers; (iii) the potential areas of misalignment between ALwO and their caregivers regarding obesity care and management [8].

The ACTION Teens study was conducted in 10 countries (including Italy) to assess ALwO, caregivers’, and HCPs’ perceptions, attitudes, and behaviors relating to obesity, plus barriers impeding effective obesity management for ALwO [8]. Analysis of global data from ACTION Teens confirmed: the need to provide adequate information regarding obesity to ALwO, caregivers, and HCPs; the presence of communication barriers between ALwO, caregivers, and HCPs; and areas of misalignment between ALwO and caregivers regarding obesity care and management [8]. Here, we report the results from an analysis of ACTION Teens participants in Italy and discuss barriers preventing ALwO from receiving effective obesity care in Italy.

## Methods

### Study design and participants

Methodology for the cross-sectional, survey-based ACTION Teens study (ClinicalTrials.gov; NCT05013359) has been reported previously [8]. 

Briefly, KJT Group Inc. (Rochester, NY, USA) collected survey responses from ALwO, caregivers, and HCPs in 10 countries (Australia, Colombia, Italy, Mexico, Saudi Arabia, South Korea, Spain, Taiwan, Turkey, and the United Kingdom) in 2021 [8]. Participants (plus parents/legal guardians of ALwO) gave informed consent to participate in the study, which was conducted in compliance with the Declaration of Helsinki, EphMRA Code of Conduct [9], and relevant regulations/laws governing data management.

In Italy, the Ethics Committee for Clinical Trials at Integrated University Hospital Verona (Verona, Italy) approved the study on August 9, 2021, and survey responses were collected from August 26 to December 13, 2021. Eligible ALwO lived in Italy, had a body mass index (BMI; calculated using self-reported weight, height, age, and sex) ≥ 95th percentile for their age and sex according to World Health Organization BMI charts [10], and were aged 12– < 18 years. Eligible caregivers resided with an ALwO in Italy ≥ 50% of the time, participated in their ALwO’s healthcare-related decisions, and were aged ≥ 25 years. HCPs had ≥ 2 years’ clinical practice experience, were practicing in Italy, spent most of their time (≥ 50%) in direct patient care, and typically saw/treated ≥ 10 ALwO per month. ALwO were excluded if they indicated that they were “extremely muscular” or had a major injury/illness that caused a significant weight change in the previous 6 months; caregivers were excluded if they responded similarly regarding their ALwO.

### Surveys

An external steering committee co-developed and approved separate surveys (with overlapping themes) for each respondent group [8].

Survey questions used single-/multiple-item selection, numeric entry fields, or Likert scales. As previously described [8], primary outcome measures included: attitudes and beliefs about obesity and its impact; weight-loss attempts, motivators, and barriers, and how successful weight loss is defined; history/frequency of weight-related conversations, who initiates weight conversations during HCP appointments, and who is responsible for initiating the conversation; interactions between HCPs and ALwO/caregivers, reasons why obesity is not discussed, and frequency of diagnosing obesity and scheduling weight-related follow-up appointments; and sources used to learn about healthy lifestyles/obesity and weight loss/management. 

### Recruitment and data collection

For Italy, the aim was to collect surveys from 650 ALwO, 650 caregivers, and 250 HCPs. This sample size was chosen to balance recruitment feasibility with statistical power.

Potential participants were identified from online databases/panels and invited to participate via email, where possible; additionally, some potential candidates (identified from databases) were contacted and screened via telephone, with eligible candidates invited to participate via email.

Caregivers were recruited from a general population sample, then asked to consent to their ALwO participating. After maximizing recruitment of “matched caregiver and ALwO pairs,” the sample size was increased by recruiting additional ALwO and caregivers. The general population sample was stratified to reflect local demographic targets gathered from government data (age, sex, region, education, and income).

Each invitation email included a unique link that was only accessible to the recipient (plus the recipient’s ALwO, if the recipient was a caregiver), which prevented unauthorized access to screening questions [8]. As ALwO and caregivers could access screening questions using the same link, response rates could not be determined for these groups.

To reduce bias, ALwO/caregiver invitation emails and screening questions were designed to conceal the topic of the study until eligibility had been confirmed. Eligible respondents could access the survey, which was provided in Italian. All respondents completed the survey online.

Decipher Survey Software (Forsta) was used to program surveys. Programming ensured that no data were missing (all questions were compulsory) and prevented “multiple participation” from respondents (surveys could not be completed more than once).

### Statistical analysis

De-identified data from all respondents with completed surveys (i.e., the full analysis set) were analyzed by KJT Group using Excel (Microsoft 365), SPSS (IBM, version 23.0), and Stata (StataCorp LLC, version IC 14.2). Data weighting was used to limit selection bias and increase the generalizability of results; weights were applied to caregiver data to reflect representative demographic targets for Italy (i.e., age, sex, region, education, and household income). All data were summarized descriptively. For continuous variables, outliers (i.e., data points two standard deviations from the mean) were removed from analyses of relevant variables; where applicable, this reduced the sample size for relevant variables.

## Results

### Participant characteristics

In Italy, 649 ALwO, 455 caregivers, and 252 HCPs were surveyed (Table 1). The response rate among HCPs was 41%; response rates for ALwO and caregivers could not be determined due to the recruitment methods utilized.

**Table 1:** Demographics and characteristics of ACTION Teens respondents from Italy

	ALwO	Caregivers	HCPs
Full Italy sample, *N*	649	455	252
Matched pairs of ALwO and caregivers, *n* (%)	32 (5)	32 (7)	N/A
Unmatched ALwO and caregivers, *n* (%)	617 (95)	423 (93)	N/A
Age in years, mean (SD)	14.0 (1.5)	43.6 (9.7)	50.8 (12.4)
Female, *n* (%)	266 (41)	196 (43)	89 (35)
BMI classification of ALwO^a^			
Obesity Class I	78% (*n* = 503)	74% (*n* = 338)	59% (SD: 20)
Obesity Class II	17% (*n* = 112)	16% (*n* = 73)	28% (SD: 13)
Obesity Class III	5% (*n* = 34)	10% (*n* = 44)	13% (SD: 10)
BMI classification of caregivers and HCPs,^b^ *n* (%)			
Underweight (< 18.5 kg/m^2^)	N/A	10 (2)	5 (3)
Healthy weight (18.5–24.9 kg/m^2^)	N/A	194 (43)	134 (69)
Overweight (25.0–29.9 kg/m^2^)	N/A	165 (36)	48 (25)
Obesity Class I–III (≥ 30.0 kg/m^2^)	N/A	86 (19)	6 (3)

Table adapted from Halford et al. [8]

*ALwO* adolescents living with obesity, *HCP* healthcare professional, *N/A* not applicable, *SD* standard deviation, *BMI* body mass index

^a^BMI classification for recruited ALwO, the ALwO of recruited caregivers, and the ALwO treated by recruited HCPs (Obesity Class I = BMI ≥ 95th percentile for age and sex; Obesity Class II = BMI ≥ 120% of 95th percentile for age and sex; Obesity Class III = BMI ≥ 140% of 95th percentile for age and sex). ALwO and caregiver data are the percentage (number) of ALwO; HCP data represent the mean percentage (SD) of their ALwO patients

^b^HCP BMI classification was based on responses from HCPs who provided data on their height and weight (excluding two outliers: *n* = 193)

Although 42% of HCPs self-reported receiving advanced training in obesity/weight management after medical school, just 17% had > 1 day of advanced training with certification/evaluation. Most HCPs were aware of clinical guidelines for treating obesity in ALwO (64%), and 86% of HCPs in this subset found the guidelines somewhat/very effective. 

### Impact of obesity

**Fig. 1:**
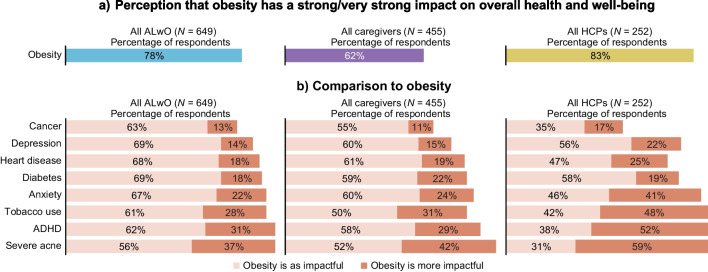
Perceived impact of obesity on overall health and well-being. Participants rated how much of an impact they thought different conditions have on a person’s overall health and well-being (1: no impact; 2: slight impact; 3: moderate impact; 4: strong impact; 5: very strong impact) (ALwO/caregiver Q510; HCP Q305). Panel **a** shows the proportion of participants who indicated obesity has a strong/very strong impact. Panel **b** shows data comparing each participant’s response about how impactful they believe obesity is in relation to the impact of other health conditions: “Obesity is more impactful” indicates a higher response for obesity than for another health condition; “Obesity is as impactful” indicates an equal response. *ALwO* adolescents living with obesity, *HCP* healthcare professional, *ADHD* attention deficit hyperactivity disorder. Figure adapted from Halford et al. [8]

The vast majority of HCPs agreed that obesity is a chronic disease (79%). Additionally, most respondents recognized that obesity has a strong impact on a person’s overall health and well-being and thought obesity was at least as impactful as cancer, heart disease, diabetes, and depression (Fig. 1).

Some ALwO and caregivers believed that a person with excess weight would find it harder to make friends (38% and 38%, respectively), do well at school (17% and 19%), and get a job (34% and 36%), relative to a person without excess weight.

Many ALwO reported that their weight often/always makes them unhappy (43%) and their body often/always makes them feel insecure (45%), although fewer caregivers reported that their child had these negative feelings (24% and 30%, respectively). 

### Perceptions of weight/health

**Fig. 2:**
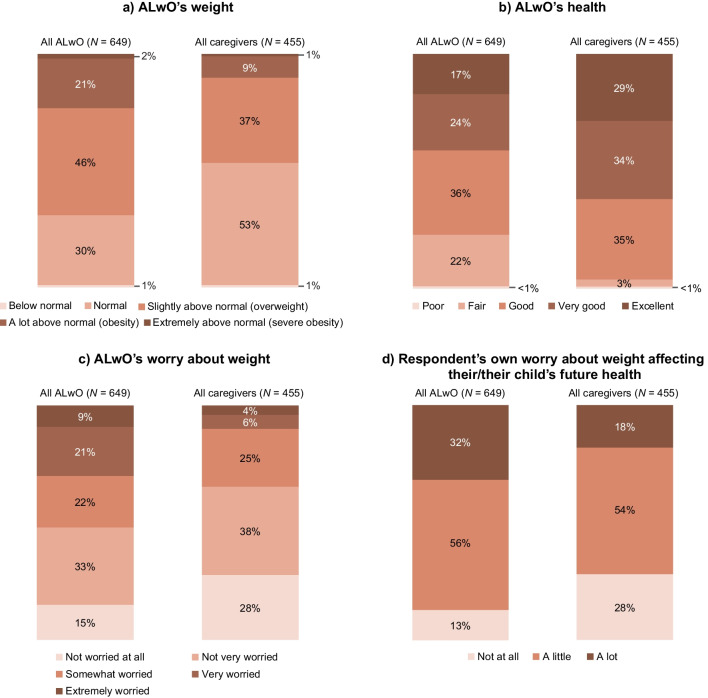
ALwO’s weight and health: perceptions and level of worry. Proportion of participants who selected each prespecified answer (ALwO Q106, Q101, Q108, and Q512; caregiver Q106, Q101, Q112, and Q515). Percentages may not sum to 100% due to rounding. *ALwO* adolescents living with obesity. Figure adapted from Halford et al. [8]

Only 46% of caregivers thought their ALwO’s weight was above normal, compared with 69% of ALwO. While 22% of ALwO felt they had fair/poor health, over half (52%) were at least somewhat worried about their weight, and the majority (87%) worried about weight affecting their future health, although the proportions of caregivers responding similarly for their ALwO were lower (3%, 34%, and 72%, respectively) (Fig. 2). Additionally, compared with HCPs, caregivers less frequently reported that their child had diagnosed comorbidities (78% of HCPs’ ALwO patients, on average, vs 33% of caregivers’ ALwO).

### Weight-loss attitudes

Most HCPs (88%) agreed that 5–10% weight loss would be extremely beneficial for an ALwO’s overall health. 

Few HCPs (11%) agreed that their ALwO patients are entirely responsible for weight loss. By comparison, a greater proportion of ALwO (69%) and caregivers (50%) agreed that they/their child were entirely responsible. 

Many ALwO (71%) and caregivers (59%) agreed that they/their child could lose weight if they really set their mind to it. However, almost half (49%) of caregivers agreed that their ALwO will naturally “slim down” as they grow older/taller, although few HCPs (17%) agreed.

For ALwO and caregivers, successful weight loss was most frequently defined as the ALwO feeling better about themselves (37% and 35%, respectively); for HCPs it was defined as improved self-esteem (58%). 

### Weight-loss motivators/barriers

**Fig. 3:**
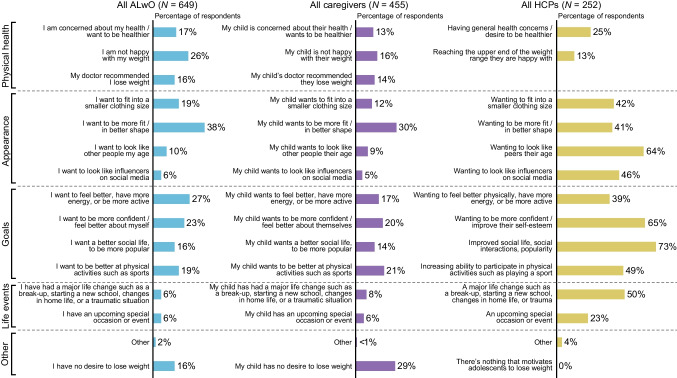
ALwO weight-loss motivators. Proportion of participants who selected each prespecified answer when asked why they/their child has wanted to lose weight (ALwO/caregiver Q208) or what most motivates adolescents to lose weight (HCP Q205). *ALwO* adolescents living with obesity, *HCP* healthcare professional. Figure adapted from Halford et al. [8]

The most frequently reported weight-loss motivator among ALwO and caregivers was the ALwO wanting to be more fit/in better shape; by contrast, HCPs thought that the top motivators were ALwO wanting to have improved social life/popularity and confidence/self-esteem, and to look like their peers (Fig. 3). Overall, only 16% of ALwO indicated they had no desire to lose weight, whereas 29% of caregivers thought their child felt this way.

**Fig. 4:**
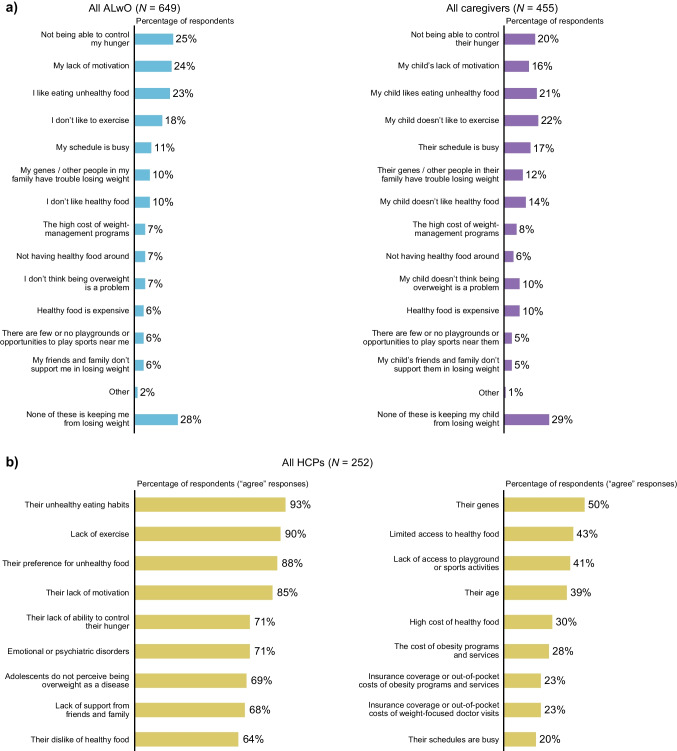
ALwO weight-loss barriers according to ALwO and caregivers (**a**) and HCPs (**b**). Proportion of participants who: selected each prespecified answer when asked what is keeping them/their child from losing weight (panel **a**: ALwO/caregiver Q210); and indicated they “strongly agree” or “somewhat agree” that each prespecified answer is a barrier to weight loss for their ALwO patients (panel **b**: HCP Q206). *ALwO* adolescents living with obesity, *HCP* healthcare professional. Figure adapted from Halford et al. [8]

The weight-loss barriers most frequently reported by ALwO were not being able to control hunger, lack of motivation, and enjoyment of unhealthy food. According to caregivers, not liking exercise, enjoying unhealthy food, and not being able to control their hunger were the most common weight-loss barriers for their ALwO (Fig. 4). Most HCPs agreed that unhealthy eating habits (93%), lack of exercise (90%), and a preference for unhealthy food (88%) were weight-loss barriers.

### Weight-loss attempts

**Fig. 5:**
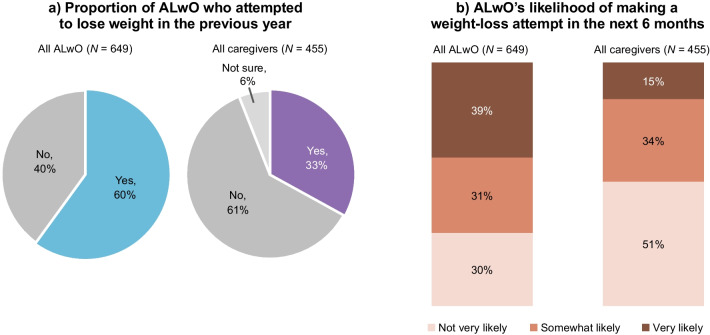
ALwO weight-loss attempts. Proportion of participants who selected each prespecified answer (panel **a**: ALwO Q108a and caregiver Q110a; panel **b**: ALwO Q109 and caregiver Q111). In panel **b**, the “very likely” category includes the answers “very likely” and “extremely likely”, and the “not very likely” category includes the answers “not very likely” and “not likely at all.” Percentages may not sum to 100% due to rounding. *ALwO* adolescents living with obesity. Figure adapted from Halford et al. [8]

HCPs thought only 41% of their ALwO patients had made a serious weight-loss attempt within the past year, with just 29% of these patients successful. A higher proportion of ALwO than caregivers reported a recent weight-loss attempt by the ALwO and that the ALwO was likely to attempt to lose weight within 6 months (Fig. 5).

### Weight-management methods

HCPs believed the most effective weight-management methods were becoming more physically active (86%), improving eating habits (79%), and reducing screen time (69%); these were also the methods that HCPs who discussed weight with ALwO most often recommended (58%, 57%, and 46% of HCPs, respectively).

The weight-management methods most frequently used by ALwO in the previous year were improving eating habits (37%) and being more physically active (30%); caregivers also reported these were their ALwO’s most commonly used methods (43% and 33%, respectively). Only 15% of ALwO said they had started a formal exercise program and only 13% had tried a specific diet/diet program; 6% had used over-the-counter weight-loss medication, 4% had taken prescription weight-loss medication, and 2% had tried weight-loss surgery.

### Weight discussions

**Fig. 6:**
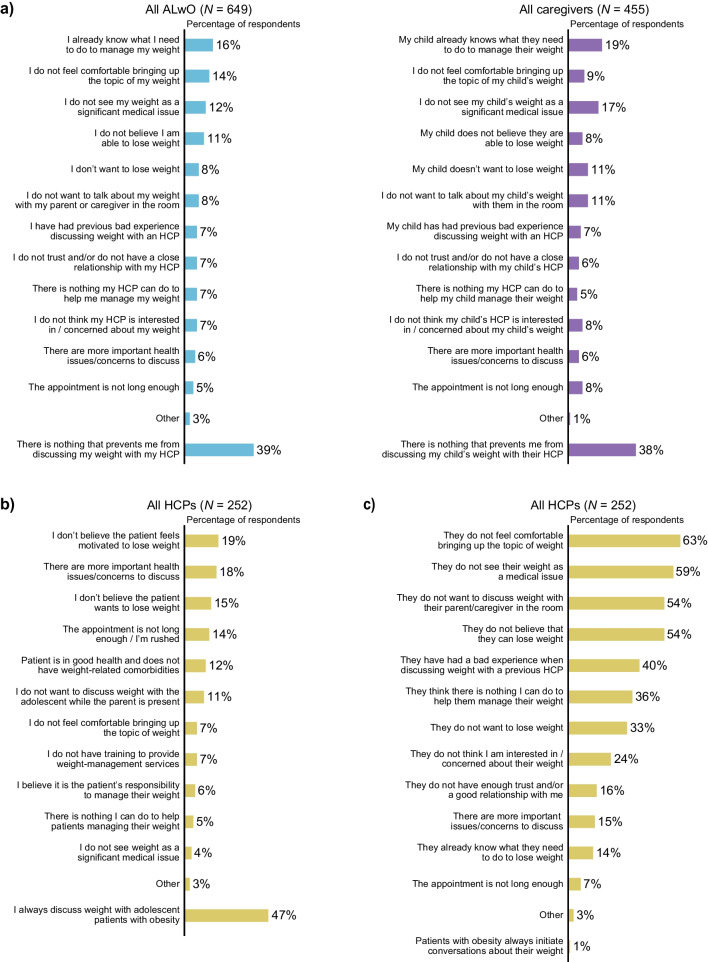
ALwO and caregivers’ self-reported barriers to discussing weight with HCPs (**a**), HCPs’ self-reported barriers to discussing weight with ALwO (**b**), and HCPs’ perception of the barriers preventing ALwO from discussing weight with HCPs (**c**). Proportion of participants who selected each prespecified answer when asked: what prevents them from discussing their/their child’s weight with their/their child’s HCP (panel **a**: ALwO/caregiver Q425); why they might not discuss weight with ALwO patients (panel **b**: HCP Q413); and why their ALwO patients might not initiate conversations about their weight (panel **c**: HCP Q412). *ALwO* adolescents living with obesity, *HCP* healthcare professional. Figure adapted from Halford et al. [8]

Although most ALwO felt they could talk honestly with their mother/father about their weight (62%), only 38% could with an HCP. Figure 6 shows barriers preventing weight discussions with HCPs.

HCPs generally reported feeling very/somewhat comfortable discussing weight with ALwO (93%). The factors that most frequently influenced whether HCPs initiate weight discussions were the patient’s mental/emotional state (62%), risk of developing new/additional obesity-related comorbidities (58%), BMI-for-age-and-sex (58%), and unhealthy lifestyle (58%).

Most ALwO (61%) and caregivers (73%) reported discussing their/their child’s weight with an HCP in the previous year; similarly, HCPs reported discussing weight with 72% of ALwO patients. Over half of ALwO (56%) and caregivers (55%) who had discussed weight with an HCP in the previous year reported that they started conversations about weight during HCP appointments, although HCPs felt they initiated discussions 55% of the time, on average. Equal proportions of ALwO thought that the responsibility for initiating weight discussions was with the HCP (44%) or themselves (44%); most HCPs (51%) thought this varied depending on the patient, although 23% felt HCPs should be responsible. 

Only 36% of ALwO and 11% of caregivers reported that they/their child had received an obesity diagnosis, despite HCPs reporting they inform 86% of their ALwO patients/caregivers about the obesity diagnosis, on average. 

### Receptiveness to HCP interactions

Among ALwO (*n* = 543) and caregivers (*n* = 367) who reported no conversations about their/their child’s weight with HCPs in the previous year or reported that the HCP was not typically the initiator of weight conversations, most would like the HCP to initiate weight discussions during appointments (ALwO: 80%; caregivers: 86%). Similarly, among ALwO (*n* = 106) and caregivers (*n* = 88) who said the HCP usually started conversations about weight in the previous year, most liked that the HCP had raised the topic (ALwO: 80%; caregivers: 90%). 

**Fig. 7:**
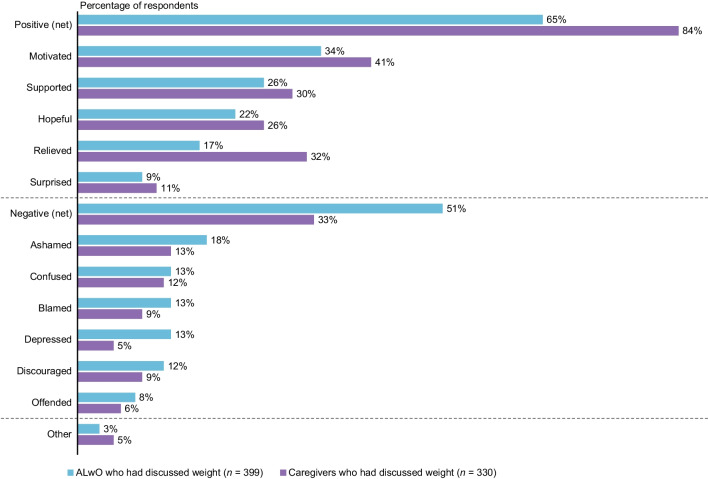
ALwO and caregivers’ feelings after their latest discussion with an HCP about weight. Proportion of participants who selected each prespecified answer (ALwO/caregiver Q410), among the subgroups of ALwO and caregivers who had discussed their/their child’s weight with an HCP in the prior year (per ALwO/caregiver Q201). The proportion of participants who selected at least one positive feeling (i.e., motivated, supported, hopeful, relieved, and/or surprised) is shown in the net positive category; the proportion of participants who selected at least one negative feeling (i.e., ashamed, confused, blamed, depressed, discouraged, and/or offended) is shown in the net negative category. *ALwO* adolescents living with obesity, *HCP* healthcare professional. Figure adapted from Halford et al. [8]

Among ALwO (*n* = 399) and caregivers (*n* = 330) who discussed the ALwO’s weight with an HCP in the previous year, most trusted the HCP’s weight-management advice (79% and 69%, respectively), agreed the HCP listened carefully when the ALwO talked about weight (78% and 67%), felt comfortable discussing weight with the HCP (72% and 67%), and agreed the HCP understood the difficulties of weight loss (74% and 61%). After their most recent weight conversations with HCPs, ALwO and caregivers had more positive than negative feelings overall, most often feeling motivated, supported, and hopeful (Fig. 7).

### Information sources

The most important and frequently used sources of information about weight management for ALwO were family and friends (most important for 15%; used by 25%) and search engines (most important for 15%; used by 24%); caregivers’ top response was doctors (most important for 26%; used by 29%). HCPs’ most commonly used information sources were medical education programs (63%) and journal articles (58%).

## Discussion

This analysis of ACTION Teens Italy data provides important insights into the barriers to effective obesity care for adolescents in Italy.

Although most ALwO surveyed in Italy were aware of the negative impact of obesity on health/well-being and correctly perceived their weight was above normal, one-third of ALwO and over half of caregivers were not aware that their/their child’s weight was above normal. This is not surprising, as previous research suggests most parents of children with obesity underestimate their child’s weight status [6]. This may be due to caregivers having an inaccurate personal reference of “weight normality” for their children [11, 12], which could lead to children acquiring the same inaccurate perception. Nevertheless, underestimation of weight status may explain why many ALwO and caregivers had not discussed the ALwO’s weight with an HCP. Additionally, caregivers’ common belief that ALwO will naturally slim down as they grow older/taller [13] may further contribute to reducing caregivers’ motivation to discuss their child’s weight with an HCP. Taken together, these findings suggest a need to provide information to caregivers about the natural course of obesity and its health implications [8].

The results of this analysis also indicate that ALwO in Italy are aware of the direct role they play in obesity management: most believed weight loss was entirely their responsibility. However, the discouraging results commonly reported following lifestyle intervention for obesity management [14] point to a need to implement techniques that engage and motivate ALwO to start and maintain a treatment program. It is therefore notable that ALwO and HCPs appeared to have differing perceptions of weight-loss motivators and barriers. For ALwO, the leading motivator was to be more fit/in better shape, whereas HCPs believed it was improving their social life. Additionally, ALwO identified their inability to control hunger as the leading barrier, but this was not among the top three barriers identified by HCPs (unhealthy eating habits, lack of exercise, and preference for unhealthy food). Of note, disrupted hunger/satiety regulation is a potent driver of body weight gain, and drugs restoring functionality of these disrupted pathways are effective in promoting weight loss [15, 16]. Therefore, increasing the ability to control hunger is likely an important treatment target. However, ALwO and HCPs’ differing perceptions of weight-loss motivators/barriers might affect weight-management strategies and reduce the chance of success. Although HCPs believed that lifestyle changes were most effective for weight management, only one in seven ALwO had tried a specific diet/diet program or started a formal exercise program, and very few had tried weight-loss medication/surgery. These findings underscore the need for further training on obesity for HCPs (as reported in recent guidelines [17]), in order to increase awareness of the biological basis of obesity [17] and the benefits of multidisciplinary treatment approaches [17, 18]. This is especially important given that only one-sixth of the HCPs surveyed had received > 1 day of advanced training in obesity with certification/evaluation.

Where misalignment was identified among ALwO, caregivers, and HCPs in Italy, lack of communication may have been a contributing factor, as many ALwO indicated they could not discuss their weight honestly with their mother/father and most could not do so with their HCP. Despite this, most ALwO would like HCPs to initiate weight discussions during appointments. Interestingly, HCPs indicated that the most important factor they consider when deciding whether to initiate weight discussions with ALwO/their caregivers is the adolescent’s mental/emotional state. Although HCPs’ attention to the psychological well-being of ALwO is important, it warrants further investigation; there is a need to determine the appropriate criteria for HCPs to consider when deciding when they should initiate weight discussions and inform ALwO/caregivers about the obesity diagnosis.

As expected, weight discussions with HCPs induced a mixture of positive and negative feelings in ALwO and caregivers, although many reported that they felt motivated, supported, and hopeful after discussing weight with the HCP. The first ALwO–HCP weight discussion may therefore play a crucial role in prompting ALwO to initiate and maintain a treatment program. This finding should encourage HCPs in Italy to be more actively involved in their patients’ weight management. Nevertheless, HCPs should not overlook the impact of negative feelings, as they could potentially discourage ALwO from discussing weight with HCPs in future. It is therefore crucial for HCPs to acquire the skills needed to motivate ALwO and have discussions about weight management in a caring, supportive, and non-judgmental way [19].

This analysis also provided further evidence that search engines are frequently used by ALwO to obtain health information [20, 21]. Unfortunately, most of the information available online is not scientifically certified and can be misleading [21]. As such, scientific societies and healthcare authorities should ensure adequate information, communicated in a simple and captivating way, is available on web channels used by adolescents.

Based on our findings, we propose several strategies to overcome difficulties in obesity management in adolescence: (i) increase awareness of the biological basis of obesity among ALwO, caregivers, and HCPs, as well as the general public and the Italian government, as there is a need for obesity to be recognized as a chronic, relapsing, and progressive disease that deserves appropriate management; (ii) increase the level of training provided to HCPs on the clinical management of obesity in children and adolescents based on the latest treatment guidelines, emphasizing the need to diagnose obesity early and initiate appropriate therapy using effective evidence-based treatments; (iii) modify the commonly held but inaccurate belief that obesity treatment is completely the responsibility of the ALwO, for example, by increasing awareness among HCPs that weight conversations with ALwO and caregivers should be initiated early and sensitively.

### Strengths and limitations

Strengths of this analysis include the use of data from ALwO, caregivers, and HCPs involved in obesity management/treatment to gain a multistakeholder perspective on adolescent obesity, and the stratified sampling method that was used to recruit ALwO and caregivers who were representative of their counterparts in the wider Italian population.

Limitations of the ACTION Teens study include the cross-sectional design and use of self-reported weight and height to calculate BMI [8]. Additionally, the HCP response rate may have reduced the representativeness of the HCPs surveyed in Italy, although the response rate in Italy was higher than in the ACTION Teens global analysis [8]. 

## Conclusion

In conclusion, we identified several potential barriers to effective obesity care for adolescents in Italy. First, the caregivers surveyed appeared to underestimate the weight of their ALwO and assumed their ALwO would naturally slim down with age, while ALwO assumed excessive personal responsibility for weight loss. This highlights a need to provide more information about obesity and its management to ALwO and caregivers. Second, the HCPs surveyed were not aligned with ALwO regarding the top weight-loss motivators and barriers, and many ALwO experienced negative feelings after discussing weight with an HCP. This suggests a need to improve the quality of communication between ALwO and HCPs, for example, by offering advanced training on obesity management to HCPs. Third, the internet appears to be a key information source for ALwO, which underscores the need for new education/communication strategies.

### What is already known on this subject?

- The prevalence of obesity among children and adolescents is high in Italy; there is an urgent need for early diagnosis and treatment.
- An international study conducted in 10 countries demonstrated there is misalignment among ALwO, their caregivers, and the HCPs who treat them regarding obesity care and management.

### What this study adds?

- This is the first comprehensive description of the perceptions, attitudes, and behaviors of ALwO, caregivers, and HCPs in Italy.
- This analysis is also the first to explore how ALwO, caregivers, and HCPs in Italy communicate regarding obesity; the results suggest a need for new strategies to improve the quality of communication.
- Finally, this analysis demonstrates there is a need to disseminate information about obesity to ALwO, caregivers, and HCPs in Italy; we recommend utilizing web-based communication channels for adolescents and offering additional training programs to HCPs.

## Data Availability

Data will be shared with bona fide researchers submitting a research proposal approved by the independent review board. Individual participant data will be shared in data sets in a de-identified and anonymized format. Data will be made available after research completion. Information about data access request proposals can be found at novonordisk-trials.com.
